# Activation of non-classical Wnt signaling pathway effectively enhances HLA-A presentation in acute myeloid leukemia

**DOI:** 10.3389/fonc.2024.1336106

**Published:** 2024-06-19

**Authors:** YuHan Ma, JunShuai Yue, Ling Gao, JingXin Zhou, Wei Chen, Jing Su, JinRong Yao, QiaoMei Shi, XiaoDong Zhao, Na Hu

**Affiliations:** ^1^ Department of Hematology, The Affiliated Suqian First People’s Hospital of Nanjing Medical University, Suqian, Jiangsu, China; ^2^ Department of Hematology, The Affiliated Hospital of Xuzhou Medical University, Xuzhou, Jiangsu, China

**Keywords:** acute myeloid leukemia, HLA-A, immune escape, non-classical Wnt signaling pathway, scRNA-seq

## Abstract

**Objective:**

The escape from T cell-mediated immune surveillance is an important cause of death for patients with acute myeloid leukemia (AML). This study aims to identify clonal heterogeneity in leukemia progenitor cells and explore molecular or signaling pathways associated with AML immune escape.

**Methods:**

Single-cell RNA sequencing (scRNA-seq) was performed to identified AML-related cellular subsets, and intercellular communication was analyzed to investigate molecular mechanisms associated with AML immune escape. Bulk RNA sequencing (RNA-seq) was performed to screen differentially expressed genes (DEGs) related to hematopoietic stem cell progenitors (HSC-Prog) in AML, and critical ore signaling pathways and hub genes were found by Gene Set Enrichment Analysis (GSEA), Gene Ontology (GO) and Kyoto Encyclopedia of Genes and Genomes (KEGG) enrichment analysis. The mRNA level of the hub gene was verified using quantitative real-time PCR (qRT-PCR) and the protein level of human leukocyte antigen A (HLA-A) using enzyme-linked immuno sorbent assay (ELISA).

**Results:**

scRNA-seq analysis revealed a large heterogeneity of HSC-Prog across samples, and the intercellular communication analysis indicated a strong association between HSC-Prog and CD8^+^-T cells, and HSC-Prog also had an association with HLA-A. Transcriptome analysis identified 1748 DEGs, enrichment analysis results showed that non-classical wnt signaling pathway was associated with AML, and 4 pathway-related genes (RHOA, RYK, CSNK1D, NLK) were obtained. After qRT-PCR and ELISA validation, hub genes and HLA-A were found to be down-regulated in AML and up-regulated after activation of the non-classical Wnt signaling pathway.

**Conclusion:**

In this study, clonal heterogeneity of HSC-Prog cells in AML was identified, non-classical wnt signaling pathways associated with AML were identified, and it was verified that HLA-A could be upregulated by activation of non-classical wnt signaling, thereby increasing antigen presentation.

## 1 Introduction

Acute myeloid leukemia (AML) is a hematologic malignancy that results from uncontrolled proliferation of hematopoietic stem cells (1). The vast majority of AML patients are elderly, with nearly a quarter of the remaining patients in children, and the 5-year overall survival, though better in pediatric patients under 5 years of age than in elderly patients, is generally low (2). Abnormal accumulation of immature myeloid cells in the patient’s bone marrow and peripheral blood is detected as the most visual clinical manifestation for the diagnosis of AML (3). However, the excessive accumulation of immature myeloid cells directly leads to bone marrow failure and peripheral blood involvement, resulting in patient death (4).

Studies have confirmed that cytogenetic variants are associated with AML pathogenesis. After delving into the genetic mechanisms, researchers have gained a deeper understanding of the histopathology, immunophenotype, and clinical heterogeneity of AML. Genetic variants that occur frequently in AML have been found to predict better disease remission and prognostic survival (5). Aberrant gene expression has revealed genetic heterogeneity in AML and is expected to be a valid biomarker for disease diagnosis and treatment (6). However, although AML is one of the malignancies with the lowest mutational burden, there are several common mutations or translocations producing immunogenic proteins that can drive malignant phenotypes (7). These mechanisms can often change the immune microenvironment in AML, and contribute to tumor immune escape. It has been reported that hematopoietic stem cell progenitor cells (HSC-Prog) present antigens via HLA molecules to promote T cell-specific recognition, thus forming an immune surveillance mechanism to prevent AML onset. However, cancer cells can drive cancer malignancy by altering the expression of antigen-presenting molecules or cytokines to reduce activation of T cells, thereby preventing harmful signals from being recognized and cleared (8). Studies have shown that activation of the classical wnt signaling pathway is related to HLA downregulation, which can lead to poor tumor antigen presentation and immune escape (9). Wnt signaling pathways are generally classified as β-Catenin-dependent (classical) and β-Catenin-independent (non-classical), which interact to maintain proliferation and developmental homeostasis of hematopoietic stem cells. The abnormal activation of Wnt/β-catenin signaling pathway is an important reason for the accumulation of leukemia stem cells, which can cause and promote AML. The non-classical Wnt signaling pathway protein (wnt5a) acts as a tumor suppressor to prevent malignant proliferation of hematopoietic stem cells (10–12). Therefore, this study will explore the relationship between non-classical wnt signaling pathways and HLA in AML.

Healthy hematopoietic stem cells (HSCs) have the ability to differentiate into bloodstream and immune cell lineages, but this ability is inhibited upon the occurrence of AML, resulting in abnormal proliferation of HSCs or HSC-Prog (13). Therefore, it is necessary to understand the differentiation direction and proliferation status of HSCs or HSC-Prog in AML. Using scRNA-seq method to analyze the cell hierarchy of bone marrow samples has become a new way to investigate the heterogeneity of AML cell clones recently (14). A report by Beneyto-Calabuig S et al. has used scRNA-seq to reveal the differentiation landscape of HSCs in AML and showed that patients were unable to produce sufficient numbers of healthy mature leukocytes due to the differentiation blockage of leukemia progenitor cells (15). In this study, referring to the recently published literature (16), scRNA-seq was performed to analyze the immune microenvironment of peripheral blood mononuclear cells (PBMCs) and identify the molecular mechanisms associated with clonal heterogeneity of leukemia progenitor cells, providing a new direction for immunotherapy in AML patients with genetic abnormalities.

## 2 Materials and methods

### 2.1 Data sources

The GSE235857 dataset was obtained from the Gene Expression Omnibus (GEO), and scRNA-seq was performed to analyze the collected PBMCs from AML patients or healthy donors, including 6 healthy samples (HL2-7: GSM7510825, GSM7510826, GSM7510827, GSM7510828, GSM7510829, and GSM7510830) and 6 AML samples (AML1-3, AML3B, and AML4-5: GSM7510831, GSM7510832, GSM7510833, GSM7510834, GSM7510835, and GSM7510836). Protein expression data of HLA-A in single cells were acquired from the Human Protein Atlas (https://www.proteinatlas.org/).

### 2.2 ScRNA-seq data analysis

The scRNA-seq datasets of 12 cases in GSE235857 were preprocessed using the Seurat (v4.3.0.1), and the single-cell datasets were debatched using the Harmony after merging the scRNA-seq datasets of 6 healthy samples and 6 AML samples. Following the normalization of the data, genes with highly variable expression from cell to cell were identified. The principal components (PCs) were subsequently calculated using the RunPCA in Seurat, and then corrected for batch effect using Harmony. The neighboring cells in the top 30 PCs were determined using the FindNeighbors function, grouped using the FindClusters function, and visualized using Uniform Manifold Approximation and Projection (UMAP). Cell types were identified based on the marker genes used for cell annotations collected from the CellMarker 2.0 database. The CellChat was used to infer the relations between ligand-receptor interactions and Intercellular communication.

### 2.3 Differential expression genes analysis

The Findmarker function was utilized to analyze the differences between the sample GSM7510831 (AML1) with the largest proportion of HSC-Prog and other samples (11 cases except GSM7510831). |Fold Change| > 1.2 and *P* < 0.05 were set as the threshold, and those meeting the conditions were defined as DEGs.

### 2.4 Gene set enrichment analysis

GSEA was performed to analyze pathways enriched by DEGs between GSM7510831 and other samples. The hub genes of critical pathway were selected for GO and KEGG enrichment analysis. A pathway of *P*<0.05 was significant.

### 2.5 Cell culture

MOLM-13 human AML cells (CL-0681), HL-60 cells (CL-0110), THP-1 cells (CL-0233) and human bone marrow HSC (CP-H262) were purchased from Procell Life Science &Technology Co., Ltd., Wuhan, China. According to the manufacturer’s instructions, MOLM-13 human AML cells (AML group) were cultured in medium containing RPMI-1640 + 10% FBS+1% P/S, and human bone marrow HSC (HL group) was cultured in human bone marrow hematopoietic stem cell complete medium (Procell, CM-H262) at 37°C in an atmosphere of 5% CO_2_. In addition, MOLM-13 human AML cells were treated with Wnt5a agonists (Foxy-5, HY-P1416, MCE) or inhibitors (Box5, HY-123071, MCE), respectively, and placed in medium culture for follow-up experiments.

### 2.6 Quantitative real-time PCR

Total RNA was extracted using Trizol reagent, according to the manufacturer’s instructions. RNA was reverse transcribed to cDNA and subsequently qRT-PCR was performed on the Applied Biosystems 7500 Fast Real-Time PCR System. Glyceraldehyde-3-phosphate dehydrogenase (GAPDH) was used as the internal control gene. The primer sequences used were shown in **Table 1**.

**Table 1:** qRT-PCR primers.

Gene	Forward (5’-3’)	Reverse (5’-3’)
ROHA	GAGCCGGTGAA ACCTGAAGA	TTCCCACGTC TAGCTTGCAG
RYK	ATTTCCTGCAC TTCACCTGG	CTTTGGCCTCC AAAAGAGTG
CSNK1D	AAGTCACGTTG TCTCGAAGCATGG	TGAAGCCAAGC CGCAAGGTAAC
NLK	ATCATCAGCACTCGCATC	GACCAGACAA CACCAAAGC
GAPDH	CATGACCACAGT CCATGCCATCACT	TGAGGTCCAC CACCTGTTGCTGTA

### 2.7 Enzyme-linked immuno sorbent assay

Protein levels of Anti-HLA Class I antibodies (abcam, ab23755) were measured using an ELISA kit that can be used to detect antigenic determinants shared by HLA-A, B, and C. The processed cells were collected, and the supernatant was taken after centrifugation, followed by the addition of biotin-labeled antibody. The horseradish peroxide conjugate was then added and incubated at 37°C for 30 min. The optical density (OD) value was measured at 450 nm with an enzyme marker to calculate the sample concentration.

### 2.8 Statistical analysis

All analyses in this study were calculated using Graphpad Prism and data were expressed as mean ± standard deviation (SD). Unpaired t-tests were used to calculate significant differences between the two groups. *P*<0.05 was statistically significant.

## 3 Results

### 3.1 AML cell data pre-processing

**Figure 1:**
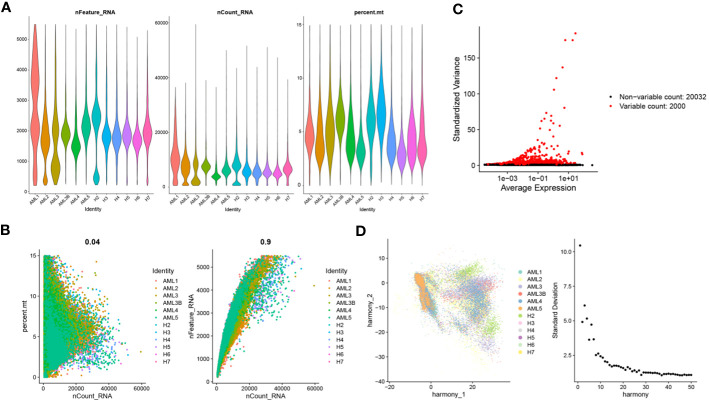
Cell quality control and classification. **(A)** Identification of 91772 cells and 22032 genes after quality control analysis of scRNA-seq for AML; **(B)** Scatterplots of the relationship between the number of sequences and the proportion of mitochondria and the number of genes before quality control, respectively; **(C)** Characteristic variance plot of gene expression profiles in the sample; **(D)** PCA results.

Ineligible cells in the GSE235857 dataset were filtered out, obtaining 91,772 cells and 22,032 genes (**Figures 1A, B**). The data were then standardized to obtain 2000 highly variable genes (**Figure 1C**). All samples and genes were processed with principal component analysis (PCA), and the top 30 PCs were selected for subsequent analysis (**Figure 1D**).

### 3.2 Identification of critical cellular subsets of AML

**Figure 2:**
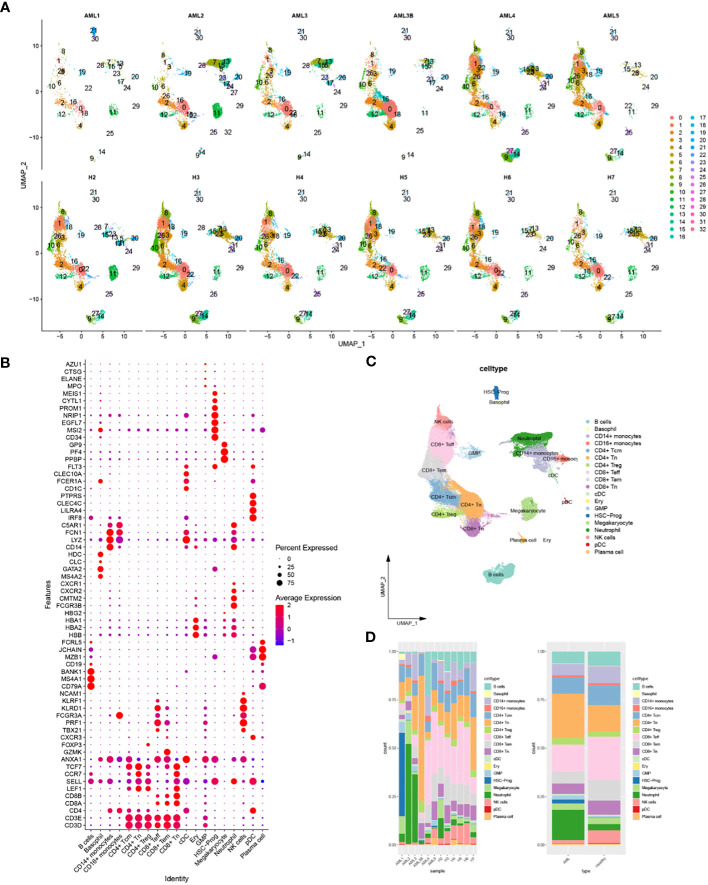
Identification of 19 cellular subsets and their intercellular communication based on scRNA-seq data. **(A)** Classification of cellular subsets in each sample; **(B)** Bubble plot of the expression level of the markers used for cell annotation; **(C)** The UMAP plot of the cell annotation results; **(D)** Stacked plot of the proportion of different cells in AML samples or healthy samples.

Cells with similar gene expression patterns were classified into one class by UMAP, identifying 33 cellular subsets. The UMAP plots demonstrated the clustering of cellular subsets in each sample, which was similar in all samples except AML1 (**Figure 2A**). The 33 cellular subsets were determined as 19 cell types by the expression level of marker genes (**Figures 2B, C**), which were B cells, Basophil, CD14^+^-monocytes, CD16^+^-monocytes, CD4^+^-Tcm, CD4^+^-Tn, CD4^+^-Treg, CD8^+^-Teff, CD8^+^-Tem, CD8+-Tn, cDC, Ery, GMP, HSC-Prog, Megakaryocyte, Neutrophil, natural killer cells, pDC, Plasma cell. Each cell had a different proportion in different samples, and this study found HSC-Prog was extremely high in AML1 but low in other samples (**Figure 2D**).

### 3.3 Cellular communication analysis of HSC-Prog signaling interactions

**Figure 3:**
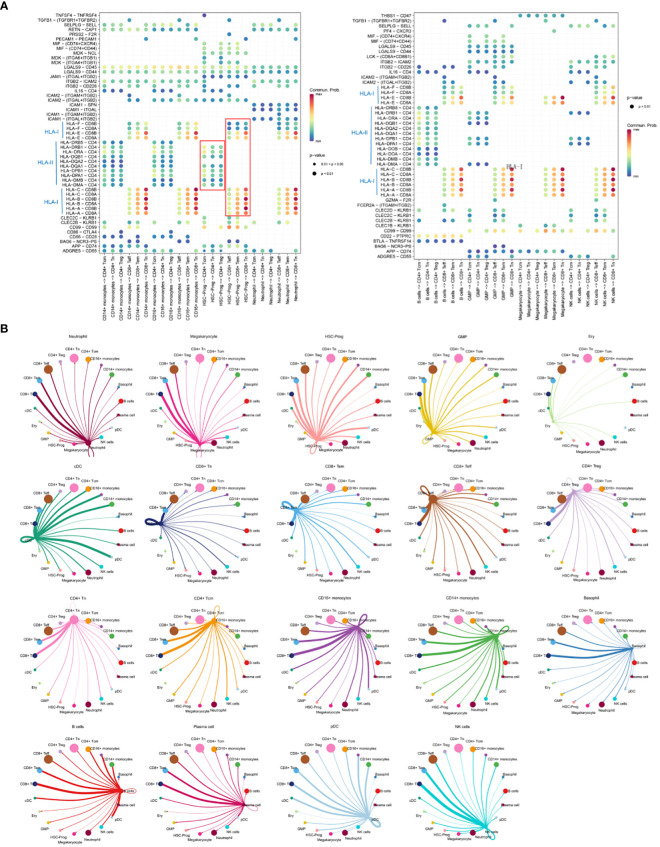
Cellular communication between HSC-Prog and other cellular subsets. **(A)** Interactions probabilities of ligand-receptor signaling associated with intercellular communication; **(B)** Cellular communication diagram.

Cellular communication analysis was performed, and focused on the communication between HSC-Prog and other cellular subsets as different ligand-receptor interaction pairs were enriched in different cellular communication. There were significant differences in the communication between HSC-Prog and CD4^+^-T or CD8^+^-T. The interaction pairs between HLA-II molecules and CD4 were only enriched in HSC-Prog-CD4^+^-T, and the probability was relatively small. However, the interaction pairs between HLA-I molecules and CD8A/B were only enriched in HSC-Prog-CD8^+^-T, and the most significant interaction pairs were HLA-A/CD8B and HLA-B/CD8B in HSC-Prog/CD8^+^-Tn (**Figure 3A**). Subsequent studies have found that there are different degrees of interaction between various cell subsets, the more significant are neutrophil-CD8 +T cells, HSC-PROg-CD8 +T cells, CD16+ monocyte-CD8 +T cells and so on. In addition, we found that HSC-Prog interacted with different CD8^+^-T or CD4^+^-T cellular subsets, but the signaling interactions between HSC-Prog and CD8^+^-T cells were stronger compared with CD4^+^-T cellular subsets (**Figure 3B**).

### 3.4 Identification of hub genes in AML cells by bulk RNA-seq

**Figure 4:**
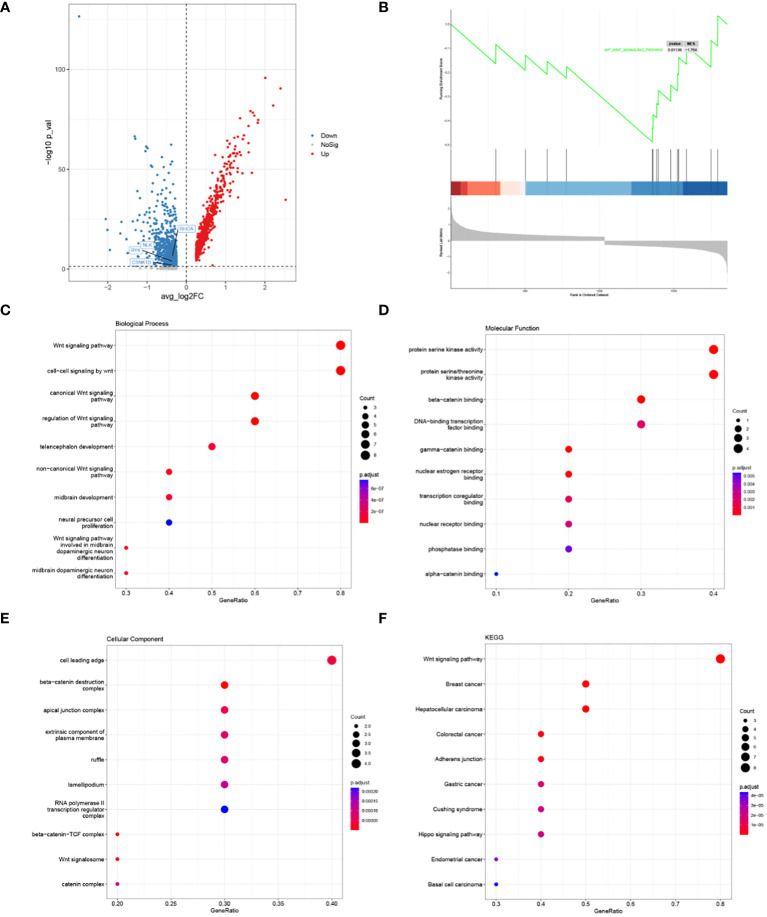
Results of HSC-Prog-based differential gene expression analysis and enrichment analysis. **(A)** Results of differential gene expression analysis of AML1 and other samples; **(B)** Enriched signaling pathways by GSEA; the top 10 hub genes; **(C)** Biological processes, **(D)** Molecular functions, and **(E)** Cellular components; **(F)** The top 10 KEGG pathways for hub genes.

A total of 1748 DEGs were identified by differential gene expression analysis, including 964 up-regulated and 784 down-regulated genes (**Figure 4A**). Subsequently, a significant signaling pathway, Wnt signaling pathway, was obtained by GSEA, and 10 hub genes associated with this pathway were obtained (**Figure 4B**). GO function and KEGG Pathway enrichment analysis were conducted based on core genes, and a significantly enriched signaling pathway was found in the biological process: non−canonical Wnt signaling pathway (**Figures 4C–F**). At the same time, we noticed that the four genes RHOA, RYK, CSNK1D and NLK in the HSC-Prog cells of the AML1 sample were significantly enriched in the non-classical Wnt signaling pathway, and as downstream genes of this pathway, the expression levels of these genes showed a significant downward trend (**Figure 4A**). It indicates that non-classical Wnt signaling pathway may be a potential key signaling pathway.

### 3.5 Activation of non-classical Wnt signaling pathway increased protein levels of HLA-A

**Figure 5:**
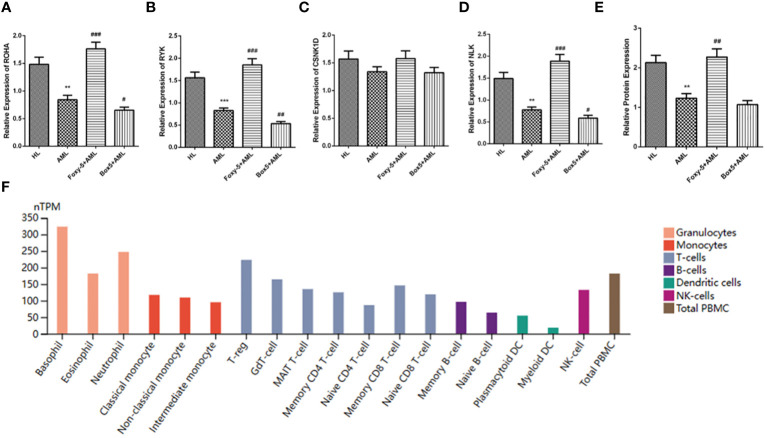
Identification of hub gene expression. **(A–D)** The relative expression levels of RHOA, RYK, CSNK1D and NLK in HL group, AML group, Foxy-5+AML group and Box5+AML group were detected by qRT-PCR; **(E)** HLA-A protein levels in HL group, AML group, Foxy-5+AML group and Box5+AML group were detected by ELISA; **(F)** Protein levels of HLA-A in various cells from bone marrow. Notes: Compared with HL, ** means *P*<0.01, *** means *P*<0.001; Compared with AML group, # means *P*<0.05, ## means *P*<0.01 and ### means *P*<0.001.

The results of qRT-PCR showed that the mRNA levels of RHOA, RYK and NLK downstream molecules of non-classical wnt signaling pathway in AML group were significantly lower than those in HL group, while the mRNA levels of CSNK1D were not significantly different. However, compared with AML group, the levels of RHOA, RYK, and NLK in Foxy-5+AML group were significantly increased, while the levels of RHOA, RYK, and NLK in Box5+AML group were not significantly different (**Figures 5A–D**). ELISA results showed that HLA-A protein level in AML group was significantly decreased compared with HL group. Compared with AML group, the protein level of HLA-A in Foxy-5+AML group was also significantly increased, but there was no significant difference in the protein level of HLA-A in Box5+AML group (**Figure 5E**). In addition, since TP53 mutations were detected in AML1 patients, to rule out the effect of this factor, we conducted experiments with non-TP53 mutated cell lines (HL-60) and TP53 mutated cell lines (THP-1). By comparing the expression of key genes and HLA-A, it was found that the occurrence of TP53 mutations was not associated with abnormal changes in the non-classical wnt signaling pathway (**Supplementary Figure 2**). **Figure 5F** presented the protein expression levels of HLA-A in various cells from the bone marrow, and it was found that the protein levels of HLA-A were generally higher in myeloid cells such as eosinophils, basophils, neutrophils, and monocytes (**Figure 5F**).

## 4 Discussion

As a rapidly progressive malignancy, AML generally presents with a deficiency of red blood cells, platelets, and granulocytes, resulting in the inability of patients to develop adequate immunity (17). Researches and advances in novel molecularly targeted therapies have brought new hope to AML patients. A number of molecules have been proved to mutate in AML and are associated with poor prognosis. Several clinical trials have indicated clinical outcomes of patients can be improved by administering molecularly targeted therapies (18–20). Besides, the administration of immune checkpoint inhibitors to prevent tumor immune escape has also become an important tool in AML treatment (21). This study focused on exploring the molecular mechanisms associated with tumor immune escape in AML, combining scRNA-seq and Bulk RNA-seq to investigate signaling with AML cells.

Resistance often occurs in the treatment of AML with immune checkpoint inhibitors, and the underlying cause of this phenomenon is that AML cells evade T cell surveillance by aberrantly regulating the antigen presentation of major histocompatibility complex (MHC)-I molecules (22). The gene cluster encoded by human MHC is known as HLA, and HLA-I molecules include HLA-A, HLA-B, and HLA-C. Cellular communication analysis of single cells reflected that HLA-A played an important role in the connection between HSC-Prog cells and CD8^+^-T cells. Peptides produced by HLA-I molecules have been reported to be recognized by tumor-specific CD8^+^-T cells, thereby activating T cells to produce immune responses. Immune checkpoint inhibitors, on the other hand, produce efficacy by increasing T cell recognition of HLA-I molecules on the tumor surface (23). However, tumors during drug resistance downregulate the HLA-I molecule-mediated endogenous antigen presentation pathway, reduce T cell infiltration, and allow tumor cells to evade host immune surveillance (24, 25). Thus, it can be determined that AML cells can inhibit the expression of HLA-A by restricting the antigen presentation pathway, thereby avoiding anti-tumor immune responses after the activation of cytotoxic CD8^+^-T cells.

Subsequently, the results of Bulk RNA-seq analysis illustrated that the non-classical Wnt signaling pathway was associated with HSC-Prog. Wnt signaling consists of one canonical cascade: the Wnt/β-catenin signaling pathway, and two atypical cascades: the planar cellular polarity (PCP) pathway and the Ca2^+^ pathway (26). In leukemia cells, β-catenin expression increases and activates the classical Wnt signaling pathway (27). When the drug resistance-related Wnt/β-catenin signaling pathway is activated, IFNγ and NFκB signaling are inhibited in tumor cells, and down-regulate the expression of MHC-I molecules (28, 29). In contrast, the atypical Wnt signaling pathway has been shown to inhibit the classical Wnt/β-catenin signaling pathway (30). Ca2^+^ signaling related Wnt5a, and PCP signaling related Frizzled-6 (Fzd6) can inhibit the expression of β-catenin (31, 32). The qRT-PCR and ELISA results of this study reflected that the hub genes RHOA, RYK, NLK and MHC-I molecules HLA-A of the atypical Wnt signaling pathway were down-regulated in leukemia cells. The mRNA levels of RHOA, RYK, NLK and HLA-A protein levels increased significantly after Wnt5a agonist treatment. In this process, the expression of CSNK1D was not greatly affected, and it was speculated that the possible reason was that CSNK1D was involved in phosphorylation of DVL, a hub gene of the non-classical Wnt signaling pathway, but without being directly regulated by the pathway (33). In conclusion, although the non-classical Wnt signaling pathway may be in an inactivated state in AML cells, the stimulation of non-classical Wnt signaling pathway may inhibit the classical Wnt/β-catenin signaling pathway, thereby increasing the expression of HLA-A.

In summary, scRNA-seq and Bulk RNA-seq methods revealed the differentiation mechanisms of leukemia cells and identified a molecular and signaling mechanism associated with leukemia immune escape. It was verified by *in vitro* experiments that stimulation of the non-classical Wnt signaling pathway may inhibit the canonical Wnt/β-catenin signaling pathway, and up-regulate the expression of HLA-A, thereby increasing T cell antigen presentation recognition. However, there are certain limitations to this study. In subsequent studies, more experiments need to be added to verify the infiltration of T cells after activation of the non-classical Wnt signaling pathway, and to deeply explore whether the hub genes of the non-classical Wnt signaling pathway can directly regulate HLA-A to increase antigen presentation.

## Data availability statement

The datasets presented in this study can be found in online repositories. The names of the repository/repositories and accession number(s) can be found in the article/**Supplementary Material**.

## Author contributions

YM: Conceptualization, Data curation, Formal analysis, Writing – original draft, Writing – review & editing. JSY: Conceptualization, Data curation, Formal analysis, Writing – original draft, Writing – review & editing. LG: Funding acquisition, Writing – original draft. JZ: Investigation, Writing – original draft. WC: Methodology, Writing – review & editing. JS: Project administration, Writing – review & editing. JRY: Methodology, Writing – review & editing. QS: Project administration, Writing – review & editing. XZ: Resources, Writing – review & editing. NH: Funding acquisition, Writing – review & editing.
