# Supplementary material for: Activation of non-classical Wnt signaling pathway effectively enhances HLA-A presentation in acute myeloid leukemia
Source: Front Oncol. 2024 Jun 19;14:1336106. doi: 10.3389/fonc.2024.1336106 (PMC11219938; doi:10.3389/fonc.2024.1336106)
Supplement: Supplementary file 1 [file DataSheet_1.docx]

S1

The bar chart showed that the proportion of HSC-Prog cells in AML1 samples was as high as 43.13%, which was significantly higher than other AML samples.


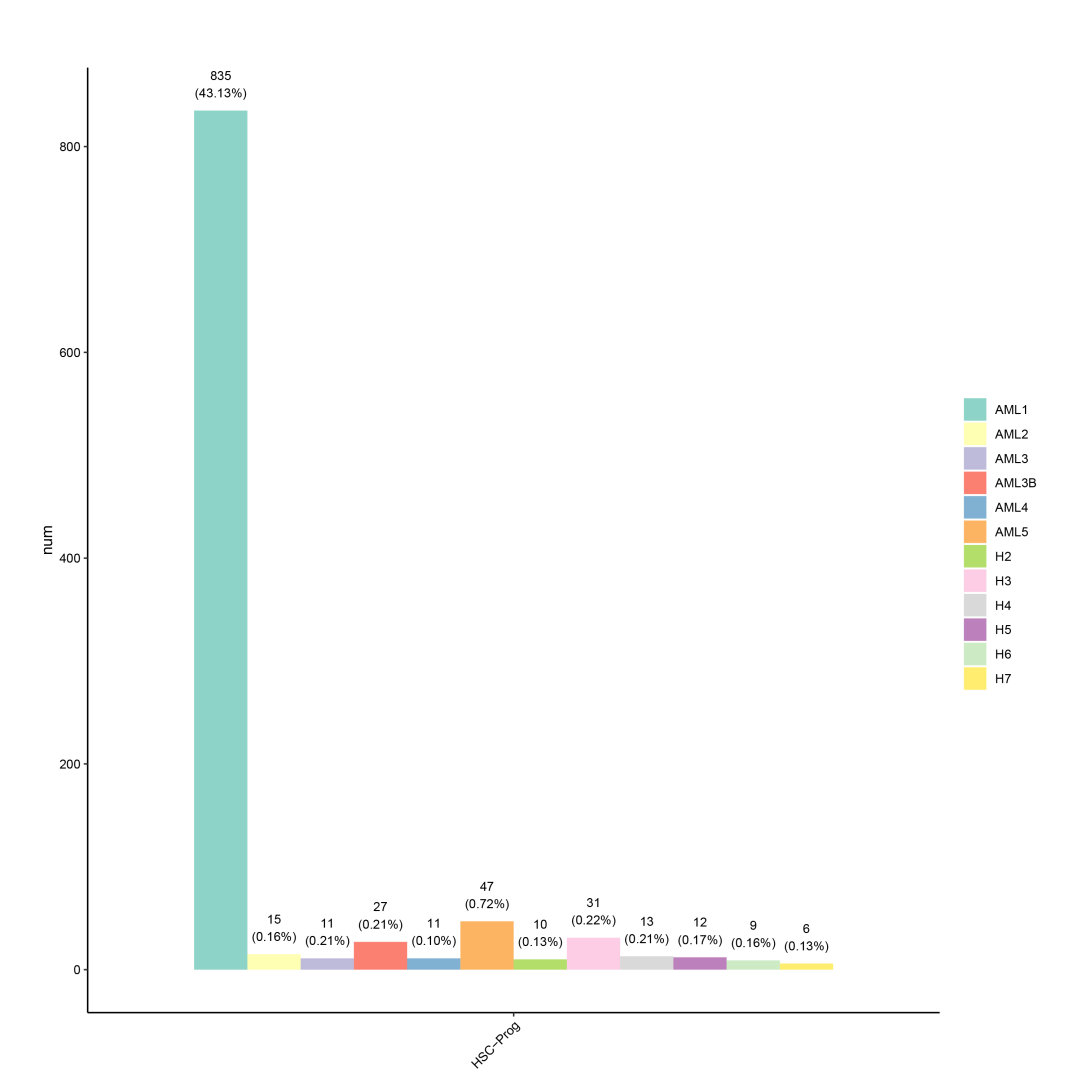


S1 Histogram of the proportion of HSC-Prog cells in each sample

S2

Since AML1 patients have TP53 mutations, we selected non-TP53 mutated HL-60 cells and TP53 mutated THP-1 cells for additional experimental validation. The results showed that mRNA levels of RHOA, RYK and NLK in AML group were significantly decreased compared with HL group, while mRNA levels of CSNK1D were not significantly different, and HLA-A protein levels were lower in the two cell lines. The levels of RHOA, RYK, NLK were significantly increased after the administration of Foxy-5, while the levels of RHOA, RYK, NLK were not significantly different after the administration of Box5 (S2-A-H). In addition, HLA-A protein levels also changed significantly after treatment with Foxy-5 or Box5 (S2 I,J).


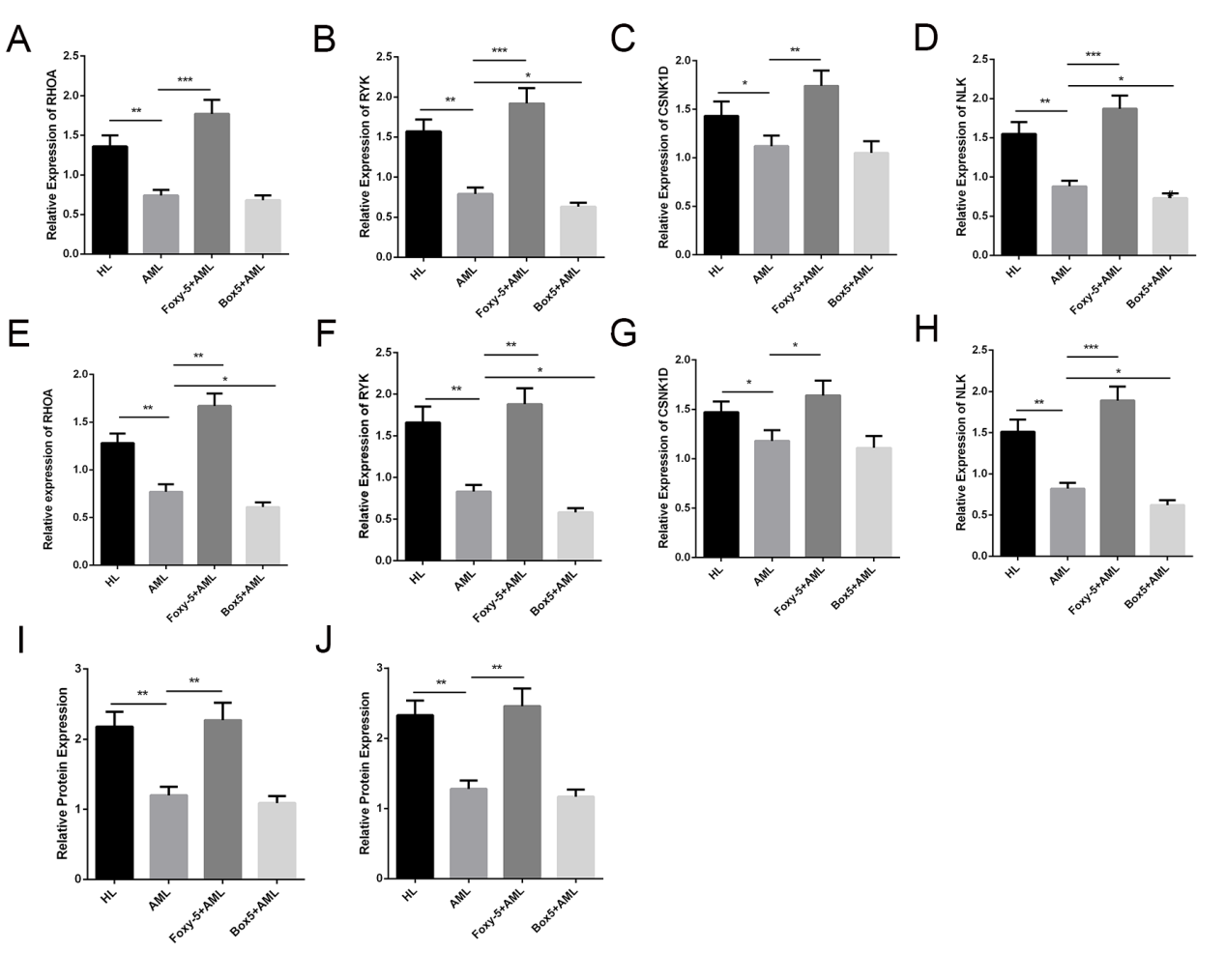


S2 Expression and identification of core genes or proteins

A-D) The relative expression levels of RHOA, RYK, CSNK1D and NLK in HL-60 cells of each group were detected by qRT-PCR; E-H) The relative expression levels of RHOA, RYK, CSNK1D and NLK in THP-1 cells were detected by qRT-PCR; I) The protein level of HLA-A in HL-60 cells of each group was detected by ELISA; J) The protein level of HLA-A in THP-1 cells of each group was detected by ELISA.
